# High Efficiency Secondary Somatic Embryogenesis in *Hovenia dulcis* Thunb. through Solid and Liquid Cultures

**DOI:** 10.1155/2013/718754

**Published:** 2013-05-29

**Authors:** Jingli Yang, Songquan Wu, Chenghao Li

**Affiliations:** ^1^State Key Laboratory of Forest Genetics and Tree Breeding (Northeast Forestry University), 26 Hexing Road, Harbin 150040, China; ^2^Key Laboratory of Nature Resource of Changbai Mountain and Functional Molecular, Ministry of Education, Yanbian University, Park Road 977, Yanji, Jilin 133002, China

## Abstract

Embryogenic callus was obtained from mature seed explants on medium supplemented with 2,4-dichlorophenoxyacetic acid. Primary somatic embryos (SEs) can only develop into abnormal plants. Well-developed SEs could be obtained through secondary somatic embryogenesis both in solid and liquid cultures. Temperature strongly affected induction frequency of secondary embryogenesis. Relatively high temperature (30°C) and germinated SEs explants were effective for induction of secondary somatic embryos, and low temperature (20°C) was more suitable for further embryo development, plantlet conversion, and transplant survival. Somatic embryos formed on agar medium had larger cotyledons than those of embryos formed in liquid medium. Supplementing 0.1 mg L^−1^ 6-benzyladenine (BA) was effective for plant conversion; the rate of plant conversion was 43.3% in somatic embryos from solid culture and 36.5% in embryos from liquid culture. *In vitro* plants were successfully acclimatized in the greenhouse. The protocol established in this study will be helpful for large-scale vegetative propagation of this medicinal tree.

## 1. Introduction

Somatic embryogenesis is the process by which somatic cells develop into plants through characteristic morphological embryo stages. Somatic embryogenesis has many potential advantages for mass propagation and genetic improvement of hardwood trees. Clonal propagation through somatic embryogenesis can shorten the time needed for breeding and can improve the uniformity and quality of nursery stock [1]. The induction of somatic embryos (SEs) requires only a single hormonal signal to induce a bipolar structure capable of forming a complete plant [2]. Secondary somatic embryogenesis is a phenomenon whereby new SEs are initiated from SEs. For some species, secondary somatic embryogenesis offers the advantages of a high multiplication rate, independence from explants source effects, and repeatability. Well-developed SEs can be obtained through secondary somatic embryogenesis on medium without plant growth regulators, as described for Rosa hybrid [3], *Chrysanthemum* [4], and *Piper nigrum* [5]. The induction of secondary SEs critically affects the rate of plant conversion. Furthermore, the embryogenesis can be maintained for a prolonged period by repeated cycles of secondary embryogenesis [6].

*Hovenia dulcis* Thunb. (Semen Hoveniae), a member of Rhamnaceae, is a woody species mainly found in China, Korea, Japan, and India [7]. The fruit, seeds, leaves, roots, and bark of* H. dulcis* are all used in traditional Chinese medicine. It is well known for treating liver diseases [8], and several studies have demonstrated that extracts of *H. dulcis* or its purified compounds can serve as detoxifying agents for alcohol poisoning [8, 9]. In addition, the wood grain is beautiful, and the wood from these hardwood trees is suitable for furniture. Traditionally, *H. dulcis* is propagated by seed or via root and wood cuttings. These methods are inefficient because seeds have impermeable seed coats that severely inhibit germination and propagation by wood and root cuttings are rather difficult to form adventitious buds or roots regeneration from cuttings.

Development of an advanced propagation technology such as somatic embryogenesis might be an efficient method for biotechnological improvement of *H. dulcis*. *In vitro* propagation of *H. dulcis* by organogenesis has been reported [10]. Our present study describes methods to obtain normal SEs and plantlet conversion following secondary somatic embryogenesis via culture of *H*. *dulcis* in agar and liquid media.

## 2. Materials and Methods

### 2.1. Somatic Embryo Induction

Seeds were collected from the mature fruit of wild *H. dulcis* in the Yangyang County of Kangwon in the republic of Korea. The seeds were first soaked in concentrated sulfuric acid for 20 min and then rinsed for 30 min with running water. The seed coats were then removed and the mature seeds soaked in 70% (v/v) ethanol for 1 min, sterilized in 2% (v/v) sodium hypochlorite solution for 20 min, and then rinsed four times with sterile distilled water.

The sterilized seeds were cultured on Murashige and Skoog (MS) [11] agar medium with 3.0% (w/v) sucrose supplemented with 1.0 mg L^−1^ 2,4-D (Sigma-Aldrich, USA). After 8 weeks, induced friable, fast-growing calli were selected and transferred to plant growth regulator-free MS agar medium containing 3% (w/v) sucrose for somatic embryo induction. During primary somatic embryogenesis, embryogenic calli frequently formed from the germinated SEs on PGR-free medium. The cultures were performed in a 250 mL Erlenmeyer flask containing 50 mL medium. All media were adjusted to pH 5.8 before adding 8 g L^−1^ plant agar (Duchefa, Haarlem, the Netherlands) and sterilized by autoclaving at 1.1 kg cm^−2^ (121°C) for 20 min. The culture room was maintained at 25 ± 1°C with a 16 h photoperiod at 36 *μ*mol·m^−2^·s^−1^ (cool white fluorescent tubes).

### 2.2. Secondary Somatic Embryogenesis from Solid and Suspension Culture

For induction of secondary somatic embryogenesis, heart-shaped, cotyledonary, and germinated primary SEs were transferred to MS agar medium containing 3% (w/v) sucrose and cultured at 20°C, 25°C, or 30°C, respectively. Eight germinated SEs were cultured per petri dish. Each experimental unit consisted of five dishes with three replicates. After 6 weeks, the percentage of secondary SE induction, number of secondary SEs per explants, and development stage of SEs were recorded. For further development, the secondary SEs were transferred to new MS agar medium containing 3.0% (w/v) sucrose and cultured at 20°C.

For secondary SE induction in suspension culture, germinated SEs were transferred to 50 mL MS liquid medium and cultured at 20°C, 25°C, or 30°C, respectively. Liquid medium was subcultured at 2-week intervals. Twenty germinated SEs were placed in each flask with 50 mL MS liquid medium by 20 replications. After 6 weeks, the induction frequency [Number of flasks formed secondary SEs/Number of flasks cultured × 100%] and fresh weight of embryogenic cell clumps in a flask were recorded. To induce secondary SE formation, embryogenic cell clumps were filtered through a 200 *μ*m stainless steel screen to remove larger clumps, and about 200 mg of cell clumps was inoculated in a flask and cultured at 20°C. After 6 weeks, the developmental stage and number of secondary SEs in a flask were recorded. To obtain the fresh weights of cell clumps and number of SEs, a 1.0 mL aliquot of the embryo suspension from each treatment was removed using a modified pipette tip (i.e., 5 mm was cut from the end of a standard 1 mL pipette tip to widen the aperture to prevent clogging), and fresh weight and number of SEs were measured. The overall number and growth of SEs were then calculated by multiplying the measured data by the total volume of the vessel medium. All cultures were agitated at 100 rpm on a gyrating shaker.

### 2.3. Plant Conversion

Germinated secondary SEs were selected and transferred to 1/3-strength MS agar medium containing 1.0% (w/v) sucrose and supplemented with 0.1 to 2.0 mg L^−1^ BA and then cultured at 20°C or 25°C. After 2 months, the percentages of plantlet conversion were recorded. Ten germinated SEs were cultured in a vessel. Each experimental unit consisted of five flasks with three replicates.

### 2.4. Transplantation

Regenerated plantlets with more than five leaves were selected and transferred to pots containing autoclaved soil mixture (1 : 3, sand : soil) in a growth chamber. Pots were covered with polythene bags to maintain high humidity and incubated in a growth chamber at 20°C or 25°C. The bags were perforated, and covers were removed after 3 weeks when the plants showed new leaves. After 2 months, the survival rate of plants was measured. Twenty plants were planted in the soil mixture (1 : 3, sand : soil), and each experiment was repeated three times. 

### 2.5. Statistical Analysis

The data variance (ANOVA) was analyzed with SPSS 16.0 for Windows (SPSS Inc., Chicago, IL, USA). Means differing significantly were compared using Duncan's multiple range test at a 5% probability level.

## 3. Results and Discussion

### 3.1. Somatic Embryo Induction

Callus was induced from seed explants after 2-week culture at 25°C and continued to grow rapidly. After 2 months in culture, yellowish-white embryogenic calli formed on the surface of the calli. The embryogenic callus induction frequency reached presumably 10% after 3 months of culture. When transferred to plant growth regulator-free agar medium, embryogenic calli differentiated into SEs, most of which could germinate but developed abnormally. These abnormal SEs rarely converted into plantlets. The appearance of abnormal embryos and subsequent low frequency of plantlet conversion are severe constraints preventing practical application of this technology [1].

### 3.2. Secondary Somatic Embryogenesis on Solid Culture

**Figure 1:**
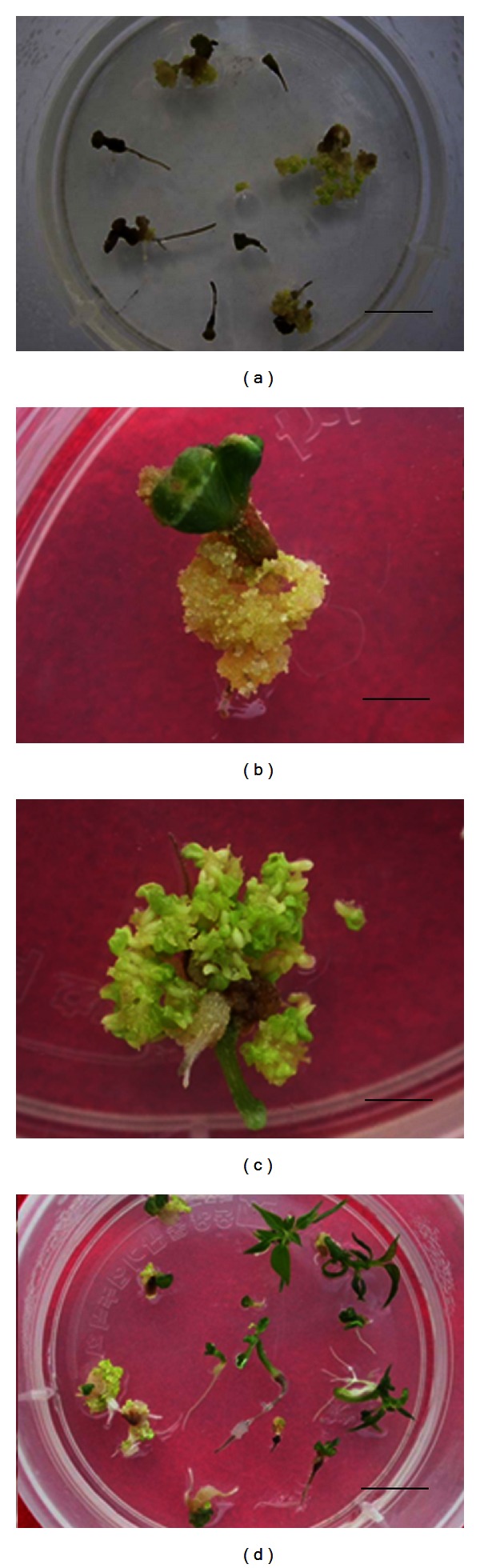
Cyclic somatic embryogenesis from germinated SEs on agar medium. (a) Embryogenic calli formed directly from germinated SEs on medium without plant growth regulators. (b) Close view of embryogenic calli formed from the primary SEs. (c) Embryogenic calli developed into cotyledonary SEs. (d) Plantlets from germinated SEs. Bars = 15 mm (a), 2 mm (b), 2 mm (c), and 15 mm (d).

Secondary SEs mainly formed from hypocotyls of germinated primary SEs after 2 weeks at 25°C (Figures 1(a) and 1(b)). The development of most of these SEs followed the normal stages of zygotic embryos from globular, heart-shaped to cotyledonary SEs and germinated (Figure 1(c)). A new cycle of secondary somatic embryogenesis initiated from primary SEs. Through this cyclic SE induction process, stable embryogenic cultures were maintained for more than 1 year. Similar observations have been reported for other species such as *Schisandra chinensis* [12, 13]. Pinto et al. [14] reported that repetitive secondary SE induction was efficient in *Eucalyptus globulus*, which produces a larger number of SEs than from primary SEs, thus increasing the potential rate of plantlet conversion. 

The temperature and developmental stage of SEs significantly affected the induction of secondary SEs. Relatively high temperature (30°C) and germinated SEs were effective for induction of secondary SEs (Table 1). At higher temperatures, more secondary SEs were induced, accomplished with delayed SE development (Table 2). Kamada et al. [15] showed similar results for cultures of early-stage somatic embryos in carrots. In general, high temperature stress can turn somatic cells into embryogenic cells [15, 16].

### 3.3. Secondary Somatic Embryogenesis in Suspension Culture

**Figure 2:**
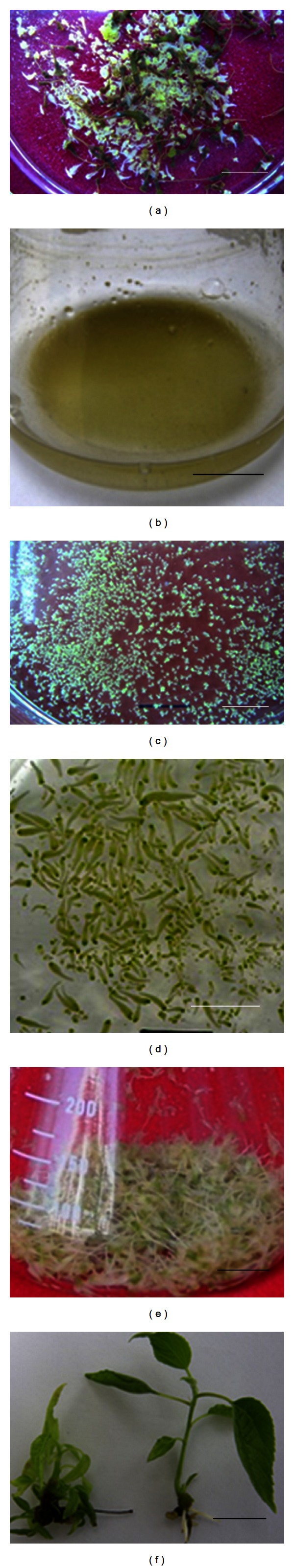
Cyclic somatic embryogenesis from germinated SEs on liquid medium. (a) Secondary SEs (bright white) induced directly from primary germinated SEs (black) in liquid medium 4 weeks after being cultured in MS liquid medium without PGRs. (b) Collected embryogenic cell clumps induced from germinated SEs in liquid medium. (c) Numerous heart-shaped SEs which developed after embryogenic calluses were cultured in MS liquid medium without PGRs for 6 weeks. (d) Cotyledonary SEs after embryogenic calluses were cultured in liquid medium for 8 weeks. (e) Germinated SEs after embryogenic calluses were cultured in liquid medium for 10 weeks. (f) Plantlets from germinated SEs on agar medium. Bars = 10 mm (a), 15 mm (b), 10 mm (c), 10 mm (d), 15 mm (e), and 10 mm (f).

To induce secondary embryogenesis from liquid cultures, germinated SEs were transferred to MS liquid medium and cultured at 20°C, 25°C, or 30°C. Numerous embryogenic cell clumps were formed from SEs after 4 weeks of culture. Similar to semisolid culture, the frequency of embryogenic cell clump formation was highest at 30°C (Table 3). These embryogenic cell clumps propagated quickly at 30°C, and their multiplication efficiency was presumably 9-fold higher than culture at 25°C (Table 3). However, no SEs developed at 25°C and 30°C. When cultured at 20°C, however, embryogenic cells developed into SEs and germinated after 4 weeks of culture (Figure 2(a)).

For synchronous development of SEs, cell suspensions were filtered through a 200 *μ*m sieve to remove larger clumps, and ~200 mg of filtered cell clumps was transferred to each flask and cultured at 20°C (Figure 2(b)). After 6 weeks, approximately 2363 SEs formed from 200 mg of cell clumps in a flask (Figure 2(c)). Our results showed that the frequency of SE formation in liquid culture was much higher than that in solid culture. The structure of cotyledonary SEs that developed in suspension culture differed somewhat from those formed in solid culture. SEs formed on solid culture had larger cotyledons and a more intact epidermis compared to embryos formed in liquid culture (Figures 2(d) and 2(e)).

### 3.4. Plantlet Conversion

Germinated SEs were transferred to 1/3-strength MS medium containing 1% (w/v) sucrose for conversion to plantlets. After 2 months of culture, the plantlet conversion frequency was examined. Most SEs could not convert into plantlets at 25°C from both agar and liquid media cultured germinated SEs (data not shown), whereas relatively low temperature (20°C) was suitable for plantlet conversion (Figures 1(d) and 2(f)). Indeed, such a low temperature is required to increase the rate of plantlet conversion [17], possibly because of a decreased content of inhibitory substances such as abscisic acid and increased levels of gibberellic acid to promote germination. Our result showed SEs culture medium significantly affected plantlet conversion (*P* < 0.001), which SEs derived from agar medium showed higher conversion frequency than that of SEs from liquid culture at the same culture conditions (Table 4). Similar results were also reported in Siberian ginseng, in which the rate of plant conversion was 97% in somatic embryos from callus culture and 76% in embryos from liquid culture [18]. This difference might be a result of the different culture conditions used during SE development.

The concentration of BA also affected plantlet conversion (*P* < 0.001), and 0.1 mg L^−1^ BA was effective for plantlet conversion to secondary SEs in both agar and liquid media (Table 4). A total of 43.3% of SEs formed from solid culture was converted to plantlets, but only 36.5% of SEs from suspension culture converted into plantlets on medium containing 0.1 mg L^−1^ BA (Table 4).

### 3.5. Transplantation

**Figure 3:**
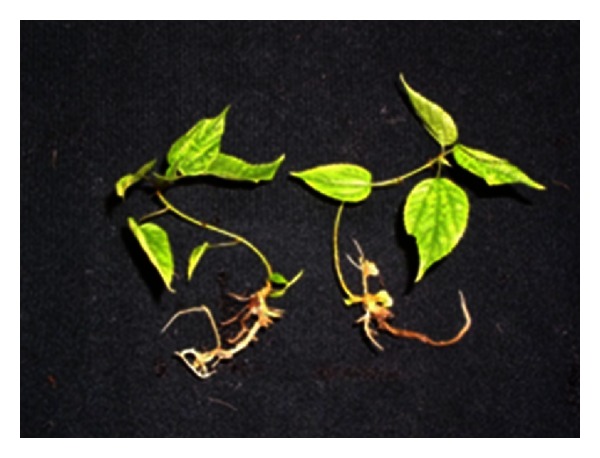
*In vitro* plantlets 2 months after acclimatization in soil. Bars = 30 mm.

When *in vitro* plantlets were cultured in the greenhouse for 4 days, new roots appeared from the plantlets cultured at 20°C. After ~10 days, new leaves began growing. The survival rate reached 82% after 2 months (Figure 3).

## 4. Conclusion

There are few reports on the effect of culture temperature on secondary SE formation and plant regeneration. The most significant result of our investigation is that a relatively high temperature (30°C) is better for induction of secondary SEs, whereas a relatively low temperature (20°C) is suitable for plantlet conversion. In conclusion, we report the optimal temperature conditions necessary for secondary somatic embryogenesis and plantlet conversion in *H*. *dulcis*. The protocol established in this study will be helpful for the conservation and large-scale vegetative propagation of *H*. *dulcis*.

## Figures and Tables

**Table 1:** Effects of temperature and developmental stage of SEs on induction of secondary SEs in *H. dulcis* after 6-week culture on MS agar medium without plant growth regulators^X^.

Temperature (°C)	Secondary SE induction frequency (%)		
	Heart-shaped SEs	Cotyledonary SEs	Germinated SEs
20	0^g^	3.3^f^	13.3^e^
25	0^g^	30.0^d^	46.7^c^
30	10.0^e^	66.7^b^	86.7^a^

^X^Values with different letters are significantly different according to Duncan's multiple range test at the 5% level.

**Table 2:** Effect of temperature on production of secondary SEs from germinated SEs of *H. dulcis* after 6-week culture on MS agar medium without plant growth regulators^X^.

Temperature (°C)	Number of secondary SEs/explant	Development stage of secondary SEs
20	5.7^c^	Cotyledonary
25	65.3^b^	Torpedo shaped
30	97.2^a^	Heart shaped

^X^Values with different letters in a column are significantly different according to Duncan's multiple range test at the 5% level.

**Table 3:** Effect of temperature on induction of embryogenic cells from germinated SEs of *H. dulcis* after 6-week culture on MS liquid medium without plant growth regulators^X^.

Temperature (°C)	Embryogenic cell induction frequency (%)^Y^	Fresh weight of embryogenic cell clumps (mg/flask)
20	0^c^	—
25	65^b^	97.3^b^
30	100^a^	894.6^a^

^X^Values with different letters in a column are significantly different according to Duncan's multiple range test at the 5% level.

^Y^Embryogenic cell induction frequency was calculated by Number of flasks formed secondary.

SEs/number of flasks cultured × 100%.

**Table 4:** Effects of BA supplementation on plantlet conversion of *H. dulcis* secondary SEs after 2 month-culture on 1/3-strength MS agar medium at 20°C^X^.

BA concentration (mg l^−1^)	Plantlet conversion frequency of secondary SEs (%)	
	SEs cultured on agar medium	SEs cultured in liquid medium
0	36.1^b^	17.0^d^
0.1	43.3^a^	36.5^b^
0.5	28.0^c^	13.3^de^
1.0	6.7^e^	0^f^
2.0	3.3^e^	0^f^

^X^Values with different letters are significantly different according to Duncan's multiple range test at the 5% level.

## Abbreviations

2,4-D: 2,4-dichlorophenoxyacetic acid
BA: 6-benzyladenine
MS: Murashige and Skoog
SE: Somatic embryo
PGR: Plant growth regulator.
